# Molecular mechanisms and cellular functions of liquid-liquid phase separation during antiviral immune responses

**DOI:** 10.3389/fimmu.2023.1162211

**Published:** 2023-05-10

**Authors:** Shuai Yang, Weishan Shen, Jiajia Hu, Sihui Cai, Chenqiu Zhang, Shouheng Jin, Xiangdong Guan, Jianfeng Wu, Yaoxing Wu, Jun Cui

**Affiliations:** ^1^ The First Affiliated Hospital of Sun Yat-sen University, Ministry of Education MOE Key Laboratory of Gene Function and Regulation, School of Life Sciences, Sun Yat-sen University, Guangzhou, Guangdong, China; ^2^ Ministry of Education Key Laboratory of Gene Function and Regulation, State Key Laboratory of Biocontrol, School of Life Sciences, Sun Yat-sen University, Guangzhou, China; ^3^ Department of Critical Care Medicine, The First Affiliated Hospital of Sun Yat-sen University, Guangzhou, China; ^4^ State Key Laboratory of Oncology in South China, Collaborative Innovation Center for Cancer Medicine, Sun Yat-sen University Cancer Center, Guangzhou, China

**Keywords:** LLPS, virus replicaiton, innate immunity, immune evasion, Viral infection disease

## Abstract

Spatiotemporal separation of cellular components is vital to ensure biochemical processes. Membrane-bound organelles such as mitochondria and nuclei play a major role in isolating intracellular components, while membraneless organelles (MLOs) are accumulatively uncovered *via* liquid-liquid phase separation (LLPS) to mediate cellular spatiotemporal organization. MLOs orchestrate various key cellular processes, including protein localization, supramolecular assembly, gene expression, and signal transduction. During viral infection, LLPS not only participates in viral replication but also contributes to host antiviral immune responses. Therefore, a more comprehensive understanding of the roles of LLPS in virus infection may open up new avenues for treating viral infectious diseases. In this review, we focus on the antiviral defense mechanisms of LLPS in innate immunity and discuss the involvement of LLPS during viral replication and immune evasion escape, as well as the strategy of targeting LLPS to treat viral infectious diseases.

## 1 Introduction

Spatiotemporal separation of cellular processes is necessary to ensure subcellular compartmentation and proper biological functions. Membrane-bound subcellular organelles are responsible for sequestering and compartmentalizing intracellular components in most cases (1); however, higher-order molecular condensates, also known as membraneless organelles (MLOs), have been recently revealed to participate in subcellular compartmentation through liquid-liquid phase separation (LLPS) (2–5). In contrast to classic organelles with lipid membranes, MLOs achieve specialized subregions through LLPS of biological polymers, such as proteins and nucleic acids, which allows subcellular enrichment of particular biomolecules to ensure their biological processes and biochemical reactions (3, 6–8). The pervasive roles of LLPS and MLOs during cellular processes have been greatly expanded in the past decade, including Cajal and promyelocytic leukemia (PML) bodies in the nucleus, as well as P-bodies (PBs) and stress granules (SGs) in the cytoplasm (9). Recent studies have focused on the particular involvement of biomolecular condensates in prompting interferon (IFN) relative antiviral biological processes (10, 11). Viruses employ strategies to form condensates for viral assembly and production, such as inclusion bodies (IBs) (12, 13). Therefore, unraveling the participation of LLPS during viral infection could open up new avenues for investigating the pathology and treatment of viral infectious diseases.

In this review, we focus on the involvement of LLPS as an antiviral defense mechanism during innate immunity and discuss the strategies involving LLPS used by viruses for replication and immune evasion. Additionally, we discuss the potential tactics of targeting the formation of LLPS to develop host-directed therapies as treatment options against viral infectious diseases.

## 2 Molecular mechanisms and cellular functions of llps condensation

The cellular membrane is responsible for dividing spaces for membranous organelles to perform specific biological functions (14–18). These membrane-bound organelles are convenient for constructing specific reaction systems and reaction environments, and reducing the influence of membrane proteins or reaction substances on the external environment. It has recently been revealed that biomolecules, including nucleic acids and proteins, can form membraneless compartments, also referred to as MLOs or LLPS (3, 19, 20). LLPS is a reversible physicochemical response in which large molecular components aggregate into a dense phase co-existing with a dilute phase. In 2009, Hyman’s team observed the formation of phase separation through the properties of P granules (21). In 2012, Li P. and colleagues found that multivalent proteins could undergo a rapid transition from small complexes to large polymeric assemblies, which increased the protein concentration (22). Moreover, Steven McKnight and colleagues found that RNA granules underwent LLPS condensate in a cell-free system (23). Since then, LLPS has become a new focus for targeting cellular processes.

As the global or regional concentration of macromolecules in solution increases, phase separation occurs from a low concentration dispersed state into a high concentration gel-like “liquid drop” state under appropriate conditions and these two states dynamically exchange. With the continued increase in the molecule concentration, the drop-like LLPS continues to transform into a colloidal form, which is termed solid-liquid phase separation. In a normal cell, the concentration of most proteins cannot reach the threshold of phase separation; however, under certain cellular processes, such as posttranslational modification, oligomerization, nucleic acid binding, or conformation change, several proteins can undergo phase separation at a low threshold concentration. In this context, cellular components can assemble more flexibly, which activates certain biochemical reactions and leads to the adoption of a continuum of material properties.

### 2.1 Feature and mechanisms of cellular LLPS condensation

**Figure 1:**
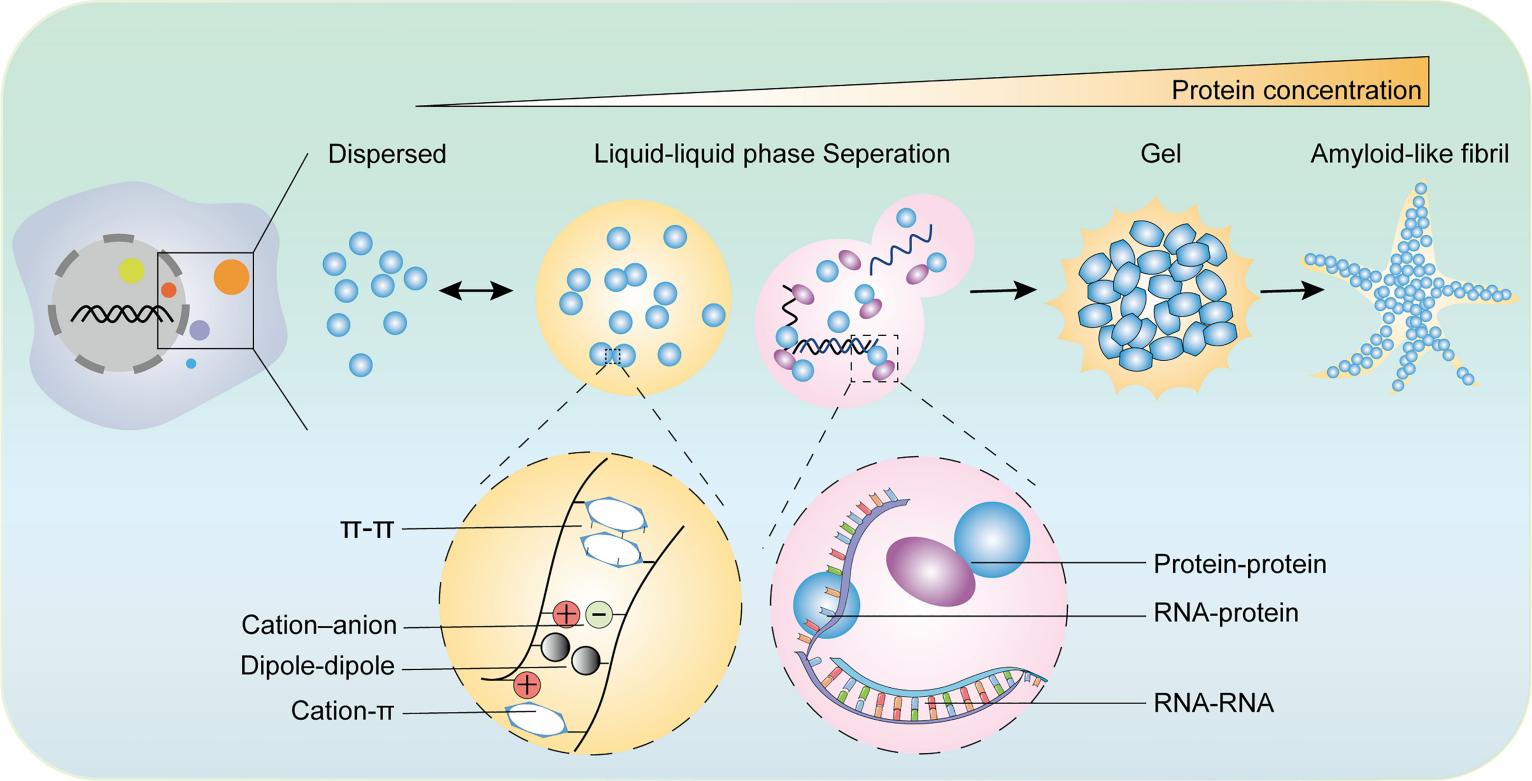
The diverse states of phase separated condensates and the driving forces of phase separation. The biomolecular condensates formed through LLPS are highly dynamic and exchange the components with surroundings. With the increase of protein concentration, the condensates of liquid-like condensates turn irreversibly to gels or amyloid fibrils. There are various types of multivalent interactions trigger phase transition including π–π interactions, cation–anion interactions, dipole−dipole interactions, cation–π interactions, and conventional multivalent interactions between protein and protein, protein and RNA, or RNA and RNA.

The LLPS system consists of two components: solutions and biomacromolecules. The multivalent forces between biomacromolecules include electrostatic interactions between charged residues, hydrogen bonds, hydrophobic interactions between weakly polar residues, superposition between aromatic residues, and cation superposition residues between positive charges and aromatic groups (3, 14, 22, 24, 25). LLPS is mainly induced by the following conditions: 1. Multivalent weak interactions between intrinsic disorder regions (IDR) or low-complexity regions of proteins (26); 2. scaffold proteins forming a phase separation through multivalent specific interaction networks, which allows enzymes or enzyme complexes to enter as “passengers”. Scaffold proteins drive LLPS and they are sufficient for spontaneous droplet and passenger molecules partition into condensates and influence the LLPS system (27); and 3. RNA-containing repetitive sequences, which are widely involved in the formation of membrane-free organelles rich in RNA/protein (28, 29). Additionally, LLPS processes are influenced by environmental parameters such as concentrations of components, the temperature of the system, salt, and pH (**Figure 1**) (30, 31).

Biomolecular condensates specially recruit certain molecules while excluding others, which enables condensates to function as selective compartments. LLPS offers a space for participants to exchange the components with surroundings. The shape of LLPS is defined by the surface tension, and the surface tension tends to reduce the area of the interface until it reaches a minimum as a spherical shape. Therefore, LLPS tends to be spherical. The spherical droplets provide the equal chemical potentials of the proteins on either side of the boundary, as the mixing tendency is offset by interaction energy (32, 33). As a result, molecules in the condensates exhibit dynamic balance (34).

Biomacromolecules in solution tend to have less free energy, meaning that they diffuse evenly in the solution. The unit concentration can be increased as an effective strategy to ensure the timely participation of macromolecules in biological functions. In solutions with higher concentrations, the additional energy output can compensate for the entropy loss caused by the aggregation of biological macromolecules (20). Once the molecule concentration increases over the critical concentration, the solution undergoes phase separation to produce a dilute solution phase or even a colloidal phase (3, 6).

LLPS can be induced by multivalent interactions of biological macromolecules, such as intracellular protein-protein, protein–RNA, and RNA–RNA interactions (3, 35). Many studies have shown that proteins containing IDRs can interact with surrounding molecules and undergo LLPS, and IDR-containing proteins are more likely to undergo LLPS (4). Typically, the IDRs are highly enriched in specific proteins containing aromatic residues, charged residues, or hydrophilic residues. The amino acids within IDRs cannot fold tightly as a stable tertiary structure, which leads to a more flexible conformation and dynamic properties. Thus, they are closer to the surroundings and have more possibilities to interact with other biological molecules (36–38). The flexible conformation in IDRs perfectly meets the requirements of multivalent weak interactions in LLPS (30, 39). In addition to LLPS mediated by IDRs, LLPS can be triggered by molecular interactions, which are theoretically stronger and more specific for the complexity of biological processes. Researchers have also reported that RNA containing a large number of repetitive sequences can trigger intranuclear phase transitions in repeat expansion disorders, which can lead to neuromuscular diseases such as Huntington’s disease, muscular dystrophy, and amyotrophic lateral sclerosis (28, 40, 41).

### 2.2 Diverse cellular functions of LLPS condensations

LLPS participates in cellular processes, including genome remapping, gene expression, and the formation of nucleosome arrays, DNA damage foci, X-chromosome inactivation (XCI) centers, paraspeckles, stress granules, proteasomes, and autophagosomes (42–45). In the nucleus, the chromatin compartments can form MLOs, in which several structural chromosomal components, such as adenosine deaminase complexing protein 1 (ADCP1), heterochromatin protein 1 α HP1a, and Chromobox 2 (CBX2), are capable of undergoing LLPS. These chromatin compartmentations have been also shown to mediate the binding of transcription factors and their DNA promoters, resulting in changes in gene transcription. Indeed, the transcription co-activators bromodomain-containing protein 4 (BRD4) and mediator complex subunit 1 (MED1) can form droplets at the super enhancer region and gather to achieve the compartmentalization of the transcription process (27). The transcriptional activation domain of the transcription factors POU class 5 homeobox 1 (OCT4) and general control transcription factor 4 (GCN4) activates gene expression through LLPS with the transcriptional mediator complex mediator (28). The transcription factor YAP can mediate gene transcription through LLPS with a PDZ-binding motif (29). HP1α has been shown to occur in LLPS in cells and *in vitro*, which could promote chromatin conformation rearrangement and increase chromatin disorders (46). In the cytoplasm, LLPS participates in a wide range of biological processes, including maintaining cellular homeostasis, controlling immune responses, and promoting inflammasome protein degradation. The key autophagy-related proteins Atg13 and Atg17 can undergo LLPS to regulate the formation of autophagy (47). Speckle-type POZ protein (SPOP), the adapter protein of the proteasome pathway, was found to target DAXX (death-domain-associated protein) through LLPS, thus mediating the proteasome pathway degradation of the target protein (48). LLPS is also believed to regulate metabolic flow (49).

Abnormal LLPS is also related to the occurrence of diseases. Indeed, LLPS of Tau has been observed in neurons of patients with amyotrophic lateral sclerosis (17), Alzheimer’s disease (AD), Parkinson’s disease (PD), and frontotemporal dementia (FTD) (40, 50–52). Meanwhile, there is emerging evidence that LLPS is involved in cancer, viral diseases, and the anti-viral infection immune response (53–56). These studies indicate that LLPS plays a vital role in human health and diseases.

## 3 LLPS condensation of viral components and their roles in pandemic virus replication

As obligatory intracellular parasites that co-evolve with their hosts over a long period, viruses have developed various strategies to drive and use LLPS in different steps of their lifecycles. Several virus-encoded proteins are characterized by a high degree of structural disorder and multivalence, as well as nucleic acid-binding capacity, which fulfills the classic prerequisites for LLPS. Viral protein-driven LLPS results in compartmentalization either in the cytoplasm or in the nucleus, and the formation of MLOs characterized by liquid-like features. These structures provide a specialized and isolated environment for the concentration of viral and cellular components to ensure the spatial organization and regulation of viral replication processes. Moreover, viral phase-separated compartments can prevent the activation of cell-intrinsic antiviral defenses by spatially excluding or sequestering innate immune components. Here, we have enumerated several examples of viral components with the capacity for LLPS and discussed their underlying mechanisms.

### 3.1 Phase-separated inclusion bodies and other forms of LLPS condensation during RNA viral replication

The biophysical mechanisms of LLPS by viral proteins concerning membraneless replication compartments have been well characterized among non-segmented negative-sense RNA viruses (nsNSV); these include several pathogens with high relevance to human diseases such as rabies virus (RABV), measles virus (MeV), respiratory syncytial virus (RSV), and Ebola virus (EBOV) (57). A defining feature of these viruses is the formation of cytoplasmic inclusions, referred to as viral inclusion bodies (IBs) or Negri bodies (NBs). FRAP experiments have revealed that viral IBs possess liquid-like properties shared by cellular MLOs, enabling them to fuse and fission, exchange materials with their surroundings, and respond to stimulation (58–60). Indeed, *in vitro* recombinant expression of viral nucleoprotein (N) and phosphoprotein (P) can reconstruct cellular minimal systems that recapitulate IB-like features (58–61). Both N and P proteins have the potential for LLPS, which is endowed with high levels of intrinsic disorder and multivalence. The RNA-binding capacity, P protein interaction, and oligomerization of N protein are essential for the IB formation of different viruses. Although the necessary structural elements of N and P vary according to virus species (as detailed in **Table 1**), the conserved IDRs in viral proteins mediate multiple protein-protein and protein-RNA interactions, which contribute to the multivalent interactions underlying LLPS (58, 60, 61, 65, 91, 92).

**Table 1:** Phase-separated viral components and their roles in viral replication.

Virus		Viral condensates	Minimal components	Required domains	Colocalizing viral components	Recruited host factors
Negative-Sense RNA Viruses	RABV	Negri bodies (NBs)	N; P (58, 61)	DD, IDD2 and PCTD of P (58)	RdRp L (62)	HSP70; FAK (63, 64)
	MeV	Inclusion bodies (IBs)	N, P (59)	PXD of P; NTAIL of N (59, 65)	RdRp L; C (66)	WDR5 (67)
	RSV		N, P (60)	oligomerization domain and C-IDR of P (60)	RdRp L; M2-1; NS2; M (68–70)	PABP; eIF4G; PP1; HSP90; HSP70 (71–74)
	EBOV		NP (75, 76)	NP-Ct and central domain (76)	RdRp L; VP30; VP35; VP24 (77)	CAD; STAU1; SMYD3; NXF1 (78–81)
Positive-Sense RNA Viruses	SARS-CoV-2		N (54, 82, 83)	the central IDR (54, 82)	RdRp L; M (54)	DDX1; G3BP1 (84, 85)
Double-Stranded DNA Viruses	KSHV	LANA NBs	LANA (86)	DNA binding domain and low complexity (LC) domains (86)		DAXX; EZH2; ORC2; histone H3K27me3 (86, 87)
	HCMV	Viral replication compartments (VRCs)	UL112-113 (88)	N-terminal oligomerization domain and C-terminal IDRs (88)		UL44 (88)
	HSV-1		ICP4 (89)	C-terminal activation domain (CTA) (89)		
	MHV-68	Virion assembly compartments (cVACs)	ORF52 (90)	N-terminal oligomerization domain and the C terminal IDR (90)		

NP, nucleoprotein.

The IBs formed *via* N-P phase separation have often been described as specialized sites for viral transcription and replication, colocalizing with the viral RNA replication machinery including N, P, and the RNA-dependent RNA polymerase (RdRp) (62, 68). Phase separation of IBs increases the local concentration of viral components to support efficient replication. In particular, the functional sub-compartments within IBs, termed IB-associated granules (IBAGs), can recruit nascent viral mRNA and viral transcription anti-terminator M2-1, with the other IB components excluded (71). IBAGs have been proposed to function as viral mRNA sorting stations, in which the nascent viral mRNAs transiently concentrate after viral transcription and replication in other areas of IBs, followed by cytosolic export with M2-1 for translation (71). The viral IBs also selectively recruit several cellular factors with identified proviral effects to support viral replication and transcription, such as FAK and hsp70 in RABV (as detailed in **Table 1**) (63, 64). Viral IBs have also been found to participate in viral RNA encapsulation and RNP formation. RNA molecules preferentially localize to N-P protein droplets and trigger the production of nucleocapsid-like structures. Accordingly, in such cases, nucleocapsid assembly is significantly improved compared to non-phase-separated conditions (65). It has also been observed that RNPs are ejected from RABV NBs in a cytoskeleton-dependent manner before being further transported to the cytoplasm (58). Moreover, LLPS constitutes an indispensable part of virus antagonism against the host’s innate defense. It has also been shown that several viral proteins recruited into IBs display an inhibitory effect on the host IFN response (66, 93). Additionally, the formation of viral inclusions can spatially exclude cellular viral sensors and sequester key factors of the downstream pathways to prevent the initiation of antiviral signaling (58, 94–96). Collectively, the formation of viral IBs by LLPS participates in different stages of the viral lifecycle.

In contrast to the members of nsNSV, influenza A virus (IAV) contains a segmented, eight-partite RNA genome that replicates in the nucleus and is encapsulated into different types of vRNP complexes. Accumulating evidence indicates that LLPS may play a vital role in the spatio and temporal control of the IAV genome assembly. Following genome replication in the nucleus, the vRNPs are exported to the cytosol and present scattered distribution in the cytoplasm, colocalizing with the cellular GTPase Rab11, which is required for the biogenesis of vRNP hotspots (97). The vRNP/Rab11 condensates constitute viral inclusions, which display characteristics of liquid organelles. The formation and maintenance of phase separated viral inclusions depended on the interactions between viral and cellular proteins, whereas the RNA–RNA intersegment interactions among different types of vRNPs appear to be independent (97). Additionally, it has been demonstrated that the formation of IAV IBs is strictly spatially regulated and highly associated with membrane-bound organelles, developing in the vicinity of the endoplasmic reticulum exits sites (ERES) and depending on ER-Golgi vesicular cycling (97). It has been proposed that IAV IBs concentrate vRNPs that are transported to the cytosol at specific sites to allow the nucleation of vRNP–vRNP interactions for viral genome assembly and posterior delivery to the plasma membrane.

Viral protein-driven LLPS also contributes to the multiplication of positive-sense RNA viruses. However, RNA viruses triggered LLPS does not involve N-P-mediated interactions. In the case of SARS-CoV-2, as a representative, the nucleoprotein alone can undergo LLPS under specific conditions, independently of other viral proteins (54, 82, 83). Resembling the phase-separating proteins of nsNSV, the SARS-CoV-2 N protein exhibits a modular architecture, comprising an ordered N-terminal domain (NTD) and a C-terminal dimerization domain (CTD) flanked by IDRs, including an N-terminal IDR, a C-terminal IDR, and a central IDR (98). The essential roles of the central IDR, which has a serine/arginine (SR)-rich region and an adjacent leucine/glutamine (L/Q)-rich region, have been widely acknowledged in N phase separation (54, 82). More importantly, it has been shown that the introduction of RNA can dramatically enhance N protein phase separation (54, 82, 83), which is independent of RNA sequence specificity (82). Further research revealed that phosphorylation in the SR-rich region is involved in regulating the RNA-induced LLPS behavior of N, including its tendency for phase separation and the viscosity of the condensates (54). Regulation by phosphorylation modification in SR region confers the N protein with dual functions during viral replication, as hyperphosphorylated N protein promotes viral transcription, and hypophosphorylated N protein mediates RNA packaging (54, 83).

N phase separation plays a general role in SARS-CoV-2 genome packaging and virion assembly. Particles with shell-like architectures have been observed in soluble N-RNA complexes *via* negative-stain electron microscopy (99). Moreover, the soluble CTD of M proteins has been shown to interact with N and induce the formation of N condensates independently of RNA (54). A mixture of three components, including N, M, and RNA, spontaneously forms mutually exclusive condensates, with a central core of N-RNA condensation surrounded by a shell of N-M, which is easily reminiscent of a virion structure (54). In addition to its role in RNA packaging, recent advances have revealed that phosphorylated N proteins facilitate the synthesis of viral RNAs through the recruitment of the cellular RNA helicase DDX1 (84). Furthermore, N-induced condensates can act to sequester the host stress granule core protein G3BP1, indicating the role of N in suppressing innate immune responses (85).

Overall, LLPS is wildly exploited by RNA viruses during infection. Viral phase-separated compartments serve as the hub for various viral processes, including genome transcription, replication, and virion assembly. Additionally, the formation of viral inclusions could also prevent the activation of cell-intrinsic defenses by spatially excluding cellular sensors and sequestering key antiviral factors. These functions suggest that targeting LLPS may be a novel effective antiviral strategy to inhibit virus replication.

### 3.2 Participation of LLPS condensation in DNA virus replication

Compared to the wide involvement of LLPS during RNA virus replication, much less is known about the roles of LLPS in the lifecycle of double-stranded DNA (dsDNA) viruses. Recent advances in the research on herpesviruses have provided new insight into the functional importance of LLPS for dsDNA viruses. Generally, Herpesviridae is a large family of DNA viruses that is divided into three subfamilies, including alpha-Herpesviridae (e.g., HSV-1), beta-Herpesviridae (e.g., HCMV), and gamma-Herpesviridae (e.g., KSHV and MHV-68) (100). In contrast to RNA viruses, herpesviruses have evolved more complex strategies in their replication cycle and progeny virion production. Following viral invasion *via* membrane fusion at the cell surface, the capsid is uncoated and transported to a nuclear pore, thus releasing viral genomes into the nucleoplasm. The invading viral genomes can either initiate transcription and replication within the nucleus during lytic infection or stay static during latent infection (100). Recent studies have revealed that LLPS plays an essential role in different stages of the herpesvirus lifecycle, including latency maintenance, genome replication, and virion assembly.

The latency-associated nuclear antigen (LANA), encoded by Kaposi’s sarcoma-associated herpesvirus (KSHV), serves as a key regulator of viral latency. During latent infection, the KSHV genome changes its chromosome conformation and forms episomes, referred to as LANA-associated nuclear bodies (LANA-NBs), in a process that is strictly dependent on LANA LLPS. LANA binds to the terminal repeats (TRs) within viral template DNA through a structured C-terminal DNA binding domain, which leads to its oligomerization and reaching a concentration threshold for the induction of phase separation. In addition to DBD, LANA contains low-complexity (LC) domains that are responsible for various interactions involved in transcription regulation (87). A combination of LANA oligomerization, driven by the KSHV DNA template, and the multivalency of N-terminal LC domains eventually contributes to the formation of LANA-NB (86). Additionally, LANA oligomerization is necessary for the recruitment of the origin recognition complex protein (ORC2), as well as other nuclear factors, including the histone H3.3 chaperone DAXX, the polycomb-associated histone H3K27me3 methylase EZH2, and the histone H3K27me3, which is thought to maintain the chromatin organization of viral episomes (87). Notably, it has been observed that LANA-NBs undergo morphological changes in association with lytic reactivation, while LLPS disruption alters the KSHV genome conformation without inducing lytic reactivation, indicating a potential association between LLPS and lytic reactivation (86). However, the involvement of LANA-mediated LLPS in the transition from the latent to the lytic stage in the viral life cycle remains to be further explained.

The lytic infection cycle is initiated by viral genome replication with the simultaneous formation of viral replication compartments (VRCs). VRCs have been described as membraneless nuclear sub-compartments that provide a pro-replicative environment for viral replication. LLPS condensations induced by viral proteins are essential for VRC formation. The human cytomegalovirus (HCMV) UL112-113 protein, comprising a conserved N-terminal oligomerization domain and C-terminal IDRs, can independently induce biomolecular condensates with liquid-like properties (88) (101). The capacity of UL112-113 to drive LLPS relies on both self-oligomerization and multivalent interactions formed by IDRs (88). Additionally, UL112-113 LLPS is crucial for the recruitment of the viral DNA polymerase accessory factor UL44 at viral genomes to facilitate their replication (88). Similarly, the ICP4 protein of herpes simplex virus type-1 (HSV-1), an essential viral transcription factor, has also been identified as an IDP with LLPS properties that drives VRC formation (89). The ICP4 C-terminal activation domain (CTA) is an indispensable structural element for LLPS, while the N-terminal activation domain (NTA) and DNA binding domain (DBD) are likely to control the protein phase behavior (89). However, the biophysical properties of HSV-1 VRCs are not fully consistent with liquid-like nature of cellular condensates, and the cellular RNA polymerase II (RNA-PolII) within HSV-1 VRCs do not follow liquid-like diffusion kinetics when crossing the condensate interface, indicating additional mechanisms involved in the compartmentalization and molecule enrichment (102). Notably, the biophysical properties of VRCs change with the progression of infection. Mature VRCs exhibit irregular shape, higher viscosity and LLPS inhibitor resistance (88, 103). This transition can be blocked by inhibiting viral DNA replication, which implicates the involvement of viral genome replication in the maturation of VRCs as a condensate (103).

Following genome replication and nucleocapsid assembly within the nucleus, virion package occurs in the cytoplasm through a complicated multistage process including tegumentation and secondary envelopment (104). The formation of cytoplasmic virion assembly compartments (VACs) has been suggested to be essential for efficient viral replication at the stage of virion assembly *via* recruiting viral tegument proteins and host vesicles containing viral glycoproteins. However, the detailed mechanisms for VAC formation have not been completely elucidated. It has been shown that the formation and maintenance of VAC of HSV-1 and HCMV depend on the remodeling of the endomembrane system (105, 106). Recent research on MHV-68 (a γ-herpesvirus) also indicated the roles of LLPS in VAC formation. MHV-68 VACs was shown to exhibit liquid-like properties (107). And ORF52, a viral tegument protein with identified LLPS properties, has been found to underlie VAC formation. Moreover, nucleic acids participate in regulation of ORF52 phase separation. During the late stage of viral replication, cytoplasmic nascent RNA was co-aggregated with ORF52, promoting LLPS of ORF52 (107).

To summarize, akin to RNA viruses, LLPS is crucially at various stages of the herpesvirus lifecycle. Specifically, the initiation of LLPS by viral proteins induces the generation of viral condensates, such as VRCs, VACs, and latency-associated nuclear bodies, thus ensuring the spatial organization and regulation of various viral processes. The fundamental mechanism appears to be conserved among herpesviruses, offering novel perspectives for antiviral therapies.

## 4 Roles of LLPS condensation during innate antiviral immunity

**Figure 2:**
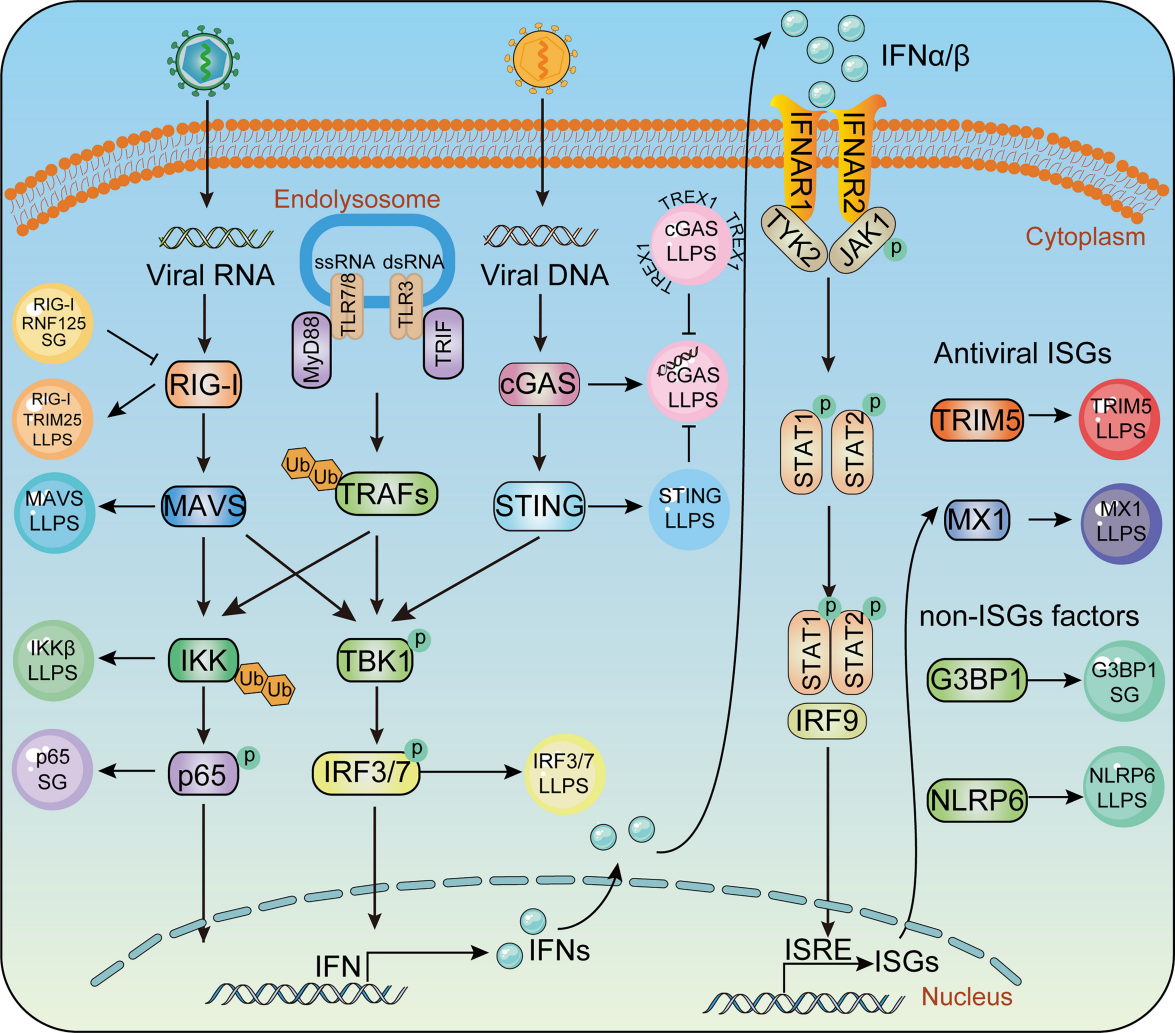
Phase separation in the innate immune pathway. Key molecules in innate immunity pathway undergoes LLPS to activate IFN signaling during virus infection, including TRIM25, MAVS, IRF3 in RLR signaling, cGAS, STING in cGAS-STING signaling and IKKβ, P65 in TLR pro-inflammatory signaling. Antiviral factors such as TRIM5, MX1, G3BP1, NLRP6 form liquid- like condensates and mediate antiviral immune responses.

The mammalian innate immune system serves as the first line of host defense. Pathogen-associated molecular pattern molecules (PAMPs) are recognized by pattern recognition receptors (PRRs) (108, 109). Upon viral infection, PRRs such as Toll-like receptors (TLRs) (110), retinoic acid-induced gene-I (RIG-I) like receptors (RLRs) (111), nucleotide-binding domain and leucine-rich repeat-containing receptors (NLRs) (112), cyclic GMP-AMP (cGAMP) synthase, and stimulator of interferon genes (STING) (113, 114), are employed to detect viral nucleic acids, hence leading to transcriptional expression of type I interferon (IFN-I) and hundreds of IFN-stimulated genes (ISGs), which amplify innate immune responses to effectively restrict viral replication (111). LLPS has also been shown to play a role in the regulation of innate immune signaling pathways (**Figure 2**). In the following section, we discuss the participation of LLPS during the innate immune response.

### 4.1 Key molecules in the RLR pathway undergo LLPS to activate IFN signaling

RIG-I is the most famous RLR which specifically recognizes viral RNA (115, 116). Upon recognition of viral RNA, RIG-I undergoes K63-linked ubiquitination, conformational change, and tetramerization, thus allowing its interaction with the adaptor protein mitochondrial antiviral-signaling protein (MAVS). MAVS then anchors to mitochondria and undergoes prion-like aggregation, which in turn recruits and promotes the phosphorylation of TANK-binding kinase 1 (TBK1) and interferon regulatory factor 3 (IRF3). Phosphorylated IRF3 undergoes dimerization and nuclear translocation, which induces the expression of type I IFN. Recently, emerging evidence has revealed that LLPS participates in several stages of the RLR signaling pathway. TRIM25, a key E3 ligase promoting K63 ubiquitination of RIG-I and RIG-I activation, can form an LLPS state with RNA through its PRY/SPRY domains (117). MAVS, a key adaptor providing a scaffold to recruit downstream TBK1/IRF3 activation in the RLR pathway, has been proven to undergo LLPS after its K63-linked ubiquitination and oligomerization. The SARS-CoV-2 N protein has been found to inhibit the K63-linked polyubiquitination of MAVS and interfere with the LLPS of MAVS, thus inhibiting the activity of MAVS to facilitate immune evasion (108). IRF3, the pivotal transcriptional factor of IFN, has also been shown to undergo LLPS with interferon (IFN)-stimulated response element DNA and compartmentalized IRF7 in the nucleus, thus inducing the transcription of IFN. Deacetylation of IRF3 mediated by deacetylase SIRT1 is considered a prerequisite for IRF3/IRF7 LLPS, as hyper-acetylated IRF3 leads to a failure of IRF3 LLPS formation, with impairment of IFN induction and increased viral load and mortality in *SIRT1* knockout mice (118). Therefore, we reason that LLPS contributes to the activation of the RLR-mediated IFN pathway.

### 4.2 Influence of LLPS in the cGAS-STING pathway

The combination of DNA with cyclic GMP-AMP synthase (cGAS) leads to the generation of a secondary messenger loop GMP-AMP (cGAMP), which activates the innate immune response. The generation of the secondary messenger cGAMP binds to STING, which leads to STING transposition to the Golgi apparatus and the induction of IFN production through TBK1 and IRF3 (40, 114, 119–121).

At the initiation of cGAS-STING signaling, the combination of DNA with cGAS strongly induces the formation of cGAS LLPS droplets. The formation of cGAS-DNA LLPS condensates is affected by the disordered and positively charged region of cGAS, as well as the DNA length (40, 122, 123). Instead of directly controlling the activation of cGAS enzymes, the cGAS-DNA phase transition is thought to enhance its capability of DNA binding by inhibiting TREX1-mediated DNA degradation (124). TREx1 mutation specifically impairs the degradation of phase-separated DNA, which is associated with the serious autoimmune disease Aicardi-Goutières syndrome (124, 125). In addition to DNA, cGAS can undergo LLPS with RNA and spermine. Although neither RNA nor spermine can induce cGAS to generate cGAMP, RNA- or spermine-enhanced formation of cGAS can enhance cGAS activity, thus leading to downstream signaling and antiviral capability (121, 126).

Moderation of cGAMP in cells can induce STING transport to the Golgi apparatus and activate the antiviral natural immune pathway (121). As cGAMP is an effective activator of STING, it needs to be degraded to ensure a controlled signal transmission theoretically. Excessive accumulation of cGAMP in cells can also induce the phase separation of STING. The STING 309-342 fragment was identified as the IDR region mediating phase separation, and two conserved amino acid mutants, E336G/E337G, in this region impair the phase separation of STING. However, the cubic membrane structure of the endoplasmic reticulum generated by STING phase separation negatively regulates STING activation by spatially isolating STING-TBK1 from the transcription factor IRF3, thus preventing innate immune over-activation. Eventually, these cubic membrane structures could be decomposed by lysosomes or autolysosomes (127). Consistent with the above results, autoimmune disease-related mutants in STING exhibit significantly reduced phase separation capacity, suggesting that the downregulation of STING phase separators plays an abnormal role under pathological conditions (127, 128).

### 4.3 Role of LLPS in the TLR signaling pathway

TLRs are the key PRRs in recognizing extracellular viral components and inducing IFN-I and pro-inflammatory cytokines. Currently, 11 TLR members have been identified in mammals, among which TLR3, TLR7, and TLR8 specifically recognize viral RNA, whereas TLR9 is a DNA recognition receptor (129). Upon activation, all TLRs, except for TLR3, recruit adaptor molecule myeloid differentiation factor 88 (MyD88) and activate transcription factors, including IRF3/7, nuclear factor-kappa B (NF-κB), and the activator protein 1 (AP-1) (130, 131). Ultimately, TLRs recognize molecular patterns leading to activation of the NF-κB.

The activation of NF-κB is a hallmark of most viral infections (132). Signaling cascades converging on the IKK complex can be activated during viral infection by viral proteins, virus-induced reactive oxygen species, and the release of viral nucleic acids. The IKK complex can induce NF-κB nuclear translocation and transcription of proinflammatory cytokines by promoting the phosphorylation and degradation of IκBα (133–136). Although the effects of NF-κB on viral replication depend on the viral species and the infected cell types, there is no dispute about the participation of NF-κB during viral infection. The IKK complex contains IKKα, IKKβ, and a regulatory subunit NEMO, which regulates the canonical NF-κB pathway. NEMO used to be considered to undergo head-to-head dimerization after binding to K63-linked or linear ubiquitination (137, 138). However, this model has been updated as NEMO has been recently shown to robustly undergo LLPS after binding to K63-linked or linear ubiquitination chains. The ubiquitin-binding (NUB) domain and the zinc-finger (ZF) domain of NEMO, which contribute to its Ub binding, are required for NEMO LLPS. Disease-associated mutations of NEMO, which impair its poly-ubiquitin binding and LLPS performance, lead to defects in NF-κB activation, indicating the importance of LLPS in NF-κB signaling (139).

### 4.4 Roles of LLPS condensation in antiviral factors

ISGs contain a large proportion of antiviral factors that play direct roles in resistance against viral infection and replication. Accumulating evidence has shown that LLPS is also involved in the direct restriction or elimination of viruses by antiviral factors. Indeed, myxovirus resistance protein 1 (MX1), one of the most well-known antiviral ISGs, can inhibit multiple viruses by blocking the early steps of the viral replication cycle (140). Mx1 has been shown to form membraneless metastable (shape-changing) condensates in the cytoplasm. The human GFP Mx1 structure in the cytoplasm is considered a phase-separated membrane-free organelle that can include the VSV nucleocapsid (N) protein (10). TRIM5α, a well-known host factor that defends against invading retroviruses such as HIV-1, can also undergo LLPS, but the association between LLPS and the antiviral capability of TRIM5α remains to be elucidated (141, 142). LLPS also participates in the antiviral processes of non-ISGs to enhance the antiviral capability of ISGs. Cellular nucleic acid-binding protein (CNBP), which directly binds to the promoter of IFNβ in response to RNA virus infection, can promote IRF3 and IRF7 binding to IFN promoters for the maximal induction of IFN production (143). CNBP binds to SARS-CoV-2 viral RNA directly and competes with N protein, which restricts the formation of N protein-RNA LLPS of SARS-CoV-2 (144). G3BP1, a member of the heterogeneous nuclear RNA-binding protein, significantly enhances the recognition of cGAS to DNA and participates in virus clearance. Further research has shown that G3BP1 pre-assembles cGAS through LLPS to promote the activity of the cGAS enzyme. Additionally, G3BP1 interacts with RIG-I to enhance its binding to dsRNA and downstream signaling pathways (145, 146). NLRP6 is central to host defense by inducing the activation of inflammatory bodies and the production of interferon (147). Several studies have shown that NLRP6 undergoes LLPS when interacting with dsRNA *in vitro* and in cell model. The intrinsic disorder poly-lysine sequence of NLRP6 is important for multivalent interaction, phase separation, and activation of inflammatory bodies. In mice, *NLRP6* deficiency or mutations in the LLPS region can lead to reduced activation of inflammasomes during infection with mouse hepatitis virus or rotavirus, indicating the anti-microbial immune function of NLRP6 LLPS (148).

Notably, m6A modification is the most common type of mRNA modification, which regulates gene expression. Three kinds of enzymes are involved in the m6A methylation of RNA: writers, erasers, and readers. Writers such as METTL3/14 and WTAP catalyze the m6A methylation of RNA, which is then recognized by readers such as YTHDF family proteins and participate in downstream translation and mRNA degradation, while erasers mediate the m6a demethylation modification. Targeting m6A not only mediates the control of the innate immune response but also blocks the replication of several viruses, including SARS-CoV-2 and influenza A virus (149, 150). The cytosolic m6A-binding proteins YTHDF1, YTHDF2, and YTHDF3 undergo phase separation (151–155); however, the association between the LLPS and the function of YTHDF proteins remains to be confirmed.

In conclusion, LLPS not only participates in the whole process of virus replication but also the antiviral process of the host cells, highlighting the importance of LLPS for viruses.

## 5 Viruses employ diverse strategies for immune evasion *via* LLPS

**Figure 3:**
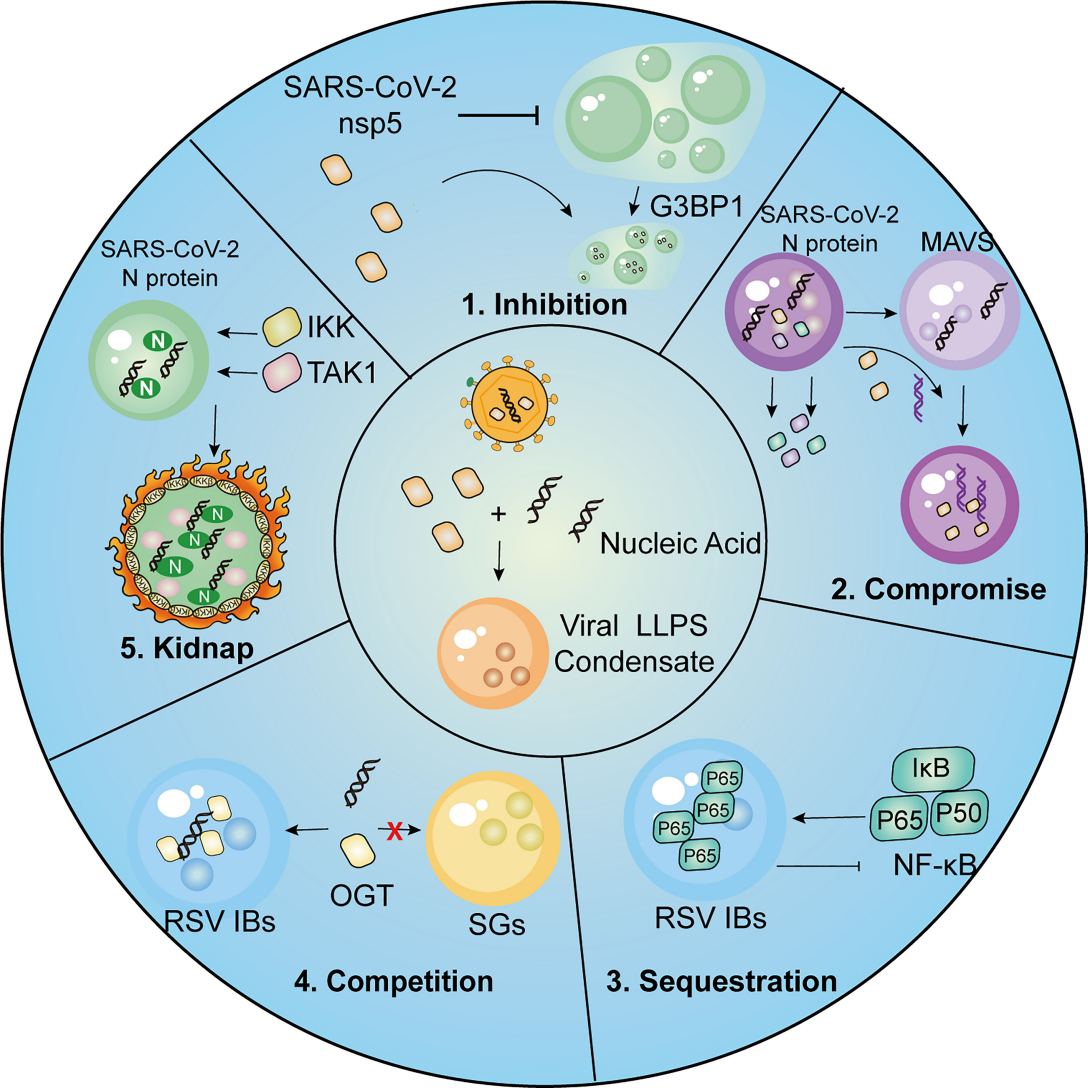
Summary of strategies employed by viruses for immune evasion.1, SARS-CoV-2 nsp5 inhibits SGs formation and promotes cleavage of G3BP1, resulting in inhibitory effect on IFN induction. 2, SARS-CoV-2 N protein undergoes LLPS thus compromising LLPS of MAVS and MAVS-dependent IFN induction. 3.The p65 subunit of NF-κB is trapped in the IBs of RSV, which leads to the restriction of its nuclear translocation and subsequent inhibitory effects on NF-κB signaling. 4. RSV-induced IBs compete OGT against SGs to suppress SG assembly. 5. SARS-CoV-2 N protein LLPS condensations recruit kinases IKKβ and TAK1 for NF-κB hyper-activation.

The preceding discussion has established the crucial role of LLPS in various aspects of the host antiviral response. To evade immune surveillance, viral proteins themselves also engage in LLPS as a means of circumventing host antiviral restrictions (**Figure 3**). This section presents a summary of several strategies used by viruses to evade host immunity *via* LLPS.

### 5.1 Viral components interrupt host antiviral LLPS condensation through direct interaction

Viral proteins directly interact with the host factors and interfere with their roles in driving LLPS, which is regarded as a fundamental mechanism underlying the activation of host antiviral immunity. In particular, SGs, serving as cytoplasmic MLOs with highly sensitive to viral infections, are frequently antagonized by viruses. The initiation of the cellular stress response requires a core protein-RNA interaction network that drives LLPS and subsequent SG assembly. To counteract this vital host immune response, viruses employ a strategy to disrupt SG formation through competitive interaction with SG core proteins and interference with their mutual aggregation.

The Ebola virus (EBOV) multifunctional protein, VP35, has been shown to disrupt the aggregation of SG proteins, which is dose-dependently induced by exogenous stress. In cases that the level of VP35 is sufficient, the oligomerization of SG components is prevented during the late stages of EBOV infection (162). Notably, VP35-mediated SG deficiency is linked to its physical interaction with SG proteins, possibly through competitive binding, rather than its ability to contend for dsRNA (162). Similarly, nsp5, the SARS-CoV-2 main protease, can interact with G3BP1, inhibiting LLPS of G3BP1 and the SG formation. Instead of the direct cleavage of G3BP1 *via* nsp5 protease activity, SARS-CoV-2 nsp5 tends to competitively combine with SG components and interrupt G3BP1-triggered LLPS (85). As SG formation serves as the signaling hub to recruit antiviral factors such as RIG-I and MAVS, restriction of G3BP1 LLPS and SG formation would downregulate the IFN induction, resulting in the inhibition of cellular antiviral responses (145, 146).

### 5.2 Formation of viral LLPS condensation impairs host LLPS and antiviral responses

In addition to direct competition with SG proteins, viral proteins can also indirectly inhibit host LLPS processes by limiting the interactions between cellular sensors and exogenous nucleic acids, which in turn allows viruses to evade and subvert the host immune surveillance and better establish infection within host cells.

Viral genomic DNA in the cytoplasm triggers the phase separation of the dsDNA sensor cGAS, which allows recognition of incoming pathogens and activation of antiviral signaling transduction. However, ORF52 and VP22, derived from KSHV and VZV, respectively, can interfere with cGAS-DNA phase separation (163). *In vitro* and in cell experiments revealed that the augment of ORF52 and VP22 displaced cGAS-DNA droplets, by simultaneously forming own liquid condensates with DNA (163). Additionally, ORF52-mediated restriction has been shown to impede the accumulation of cGAS substrates (ATP and GTP), thereby disrupting cGAS-induced signaling transduction (163). The capability of viral proteins to form multivalent interactions with DNA and undergo DNA-induced condensation plays an essential role in ORF52-mediated or VP22-mediated restriction of cGAS-DNA phase separation, which is determined by their IDRs (163). Another viral tegument protein, ORF9, from VZV, disrupts cGAS–DNA oligomers by undergoing DNA-dependent LLPS without associating with cGAS, which is similar to ORF52 and VP22 (163, 164).

Interestingly, RNA viruses such as SARS-CoV-2 could also impair MAVS function by restricting the formation of MAVS LLPS (159). *In vitro*, with the accumulation of N protein, the MAVS droplets are gradually displaced by N protein droplets, which relies on the dimerization domain of SARS-CoV-2 N protein (159). In addition to disrupting MAVS LLPS, SARS-CoV-2 N protein has been shown to directly interact with MAVS and inhibit Lys63-linked poly-ubiquitination and aggregation of MAVS, indicating multiple mechanisms by which SARS-CoV-2 can inhibit the MAVS-mediated interferon response *via* N protein (159).

### 5.3 Viral LLPS condensation sequesters host antiviral molecules to minimize IFN antiviral responses

Another evasion strategy that is widely employed by viruses is to sequester and shield key antiviral signaling molecules of the innate immune system within viral inclusions to ensure effective evasion and suppression of host innate immunity.

Respiratory syncytial virus (RSV) N protein within viral inclusion bodies (IBs) has been shown to sequester critical antiviral signaling molecules such as MAVS and MDA5, thus leading to the significant inhibition of RSV infection-induced IFN responses (94). The sequestration of MAVS and MDA5 by phase-separated N protein is further evidenced through their re-localization within IB-like structures by introducing RSV N and P proteins (94). Similarly, recruitment of the NF-κB subunit p65 within RSV IBs hinders its activation and subsequent nuclear translocation, thereby inhibiting downstream NF-κB signaling (165). RSV infection could also result in the sequestration of MAPK p38 within IBs and the subsequent interference with signal transduction through MAPK/MK2, which could be beneficial for virus replication (96). RABV multifunctional P protein targets cellular STAT proteins and sequesters them in the cytoplasm, which prevents the IFN-induced nuclear translocation of STATs and altogether inhibits JAK-STAT signaling in RABV-infected cells (166, 167). Similarly, infection with SFTSV leads to re-localization of the key signaling molecules to viral IBs, such as TBK1/IKKϵ and IRF3/IRF7 (168, 169).

The formation of viral IBs mediated by viral protein-driven LLPS results in spatial isolation of critical antiviral molecules from the cytoplasmic environment, leading to the blockage of host innate immunity.

### 5.4 Viral LLPS condensation competitively interacts with host molecules for IFN restriction

Activation of antiviral responses requires the mobilization of various cellular resources. Therefore, viruses have also developed a novel mechanism for evading the immune system by competing with host cells for the cellular components of the antiviral immune system, which relies on viral proteins that promote phase separation.

As a concrete example, a KSHV inhibitor of cGAS (KicGAS) encoded by ORF52, forms condensates upon interacting with cytoplasmic dsDNA, thereby competitively inhibiting DNA-induced phase separation and subsequent activation of the dsDNA sensor cGAS (170). The N-terminal ordered domain has been shown to mediate KicGAS self-oligomerization, while the C-terminal IDR presumably mediates collective multivalent interactions with DNA. Both the N- and C-terminal domains of KicGAS are essential for KicGAS phase separation with DNA and the inhibition of cGAS (170).

Additionally, viral inclusions spatially separate cellular components from the cytoplasmic environment and preclude their roles in the cellular stress response, which presents a novel mechanism by which viruses contend for and occupy cellular resources to counteract antiviral immunity. For instance, the O-linked N-acetylglucosamine (OGN) transferase participates in regulating the stress response by catalyzing the post-translation modification of ribosomal proteins and supporting SG assembly (171, 172). It has been found that RSV infection results in the accumulation of OGT within viral inclusion bodies, leading to the inhibition of SG formation (96). Similarly, numerous necessary components for SG formation, including eIF4G, eIF3, PABP, and G3BP1, are also isolated within EBOV inclusions, which impedes the virus-induced SG assembly in EBOV-infected cells (162). Interestingly, the sequestration of these cellular proteins as well as the blockage of SG formation could be partly reversed upon stimulation of oxidative stress, with clear shrinkage and fragmentation of the viral IBs (96, 162), suggesting that sequestered proteins may be released into the cytoplasm due to the interruption of IB formation in response to exogenous stress. However, how viruses achieve the plunder of host factors within viral inclusions remains to be clarified as the formation of viral inclusion-like structures of nucleocapsid proteins was found to be insufficient to induce the relocation of SG proteins (162). In consideration of the RNA-binding capacity of SG proteins, the viral RNA colocalized with inclusion-bound granules may play a role in their competition (162).

### 5.5 Viral LLPS hijack host factors for viral replication and IFN blockade

Viruses hijack functional host factors and redirect them to virus-induced inclusions to implement their roles in viral life cycle replication, which is usually dependent on the interaction between host factors and phase-separating viral proteins.

It has been shown that hsp70, the major cellular heat shock protein, is frequently recruited by viruses and plays an indispensable role in various steps of viral replication. Indeed, RABV infection has been shown to induce enhanced expression of hsp70 and its recruitment to NBs, where viral transcription and replication occur (63). In contrast, specific inhibition of hsp70 synthesis significantly impaired viral transcription, viral protein accumulation, and virion production, indicative of a proviral effect of Hsp70 during RABV infection (63). Likewise, EBOV NP is known to recruit the cellular factor CAD into IBs, providing pyrimidines for EBOV RNA synthesis (78). The N protein of SARS-CoV-2 can also undergo liquid-liquid phase separation and recruit TAK1 and IKK complex, the key kinases of NF-κB signaling, which leads to the hyperactivation of NF-κB pro-inflammatory responses (173).

In addition to hijacking host factors to assist in viral replication, phase-separated viral proteins also recruit host-negative regulators to counteract host immune responses. For example, MeV-induced cytoplasmic IBs recruit WD repeat-containing protein 5 (WDR5) and promote viral replication (67). Depletion of WDR5 has been shown to enhance the induction of IFN-β during MeV infection (67). Additionally, the Tax1-binding protein 1 (TAX1BP1), which is directly involved in the negative regulation of NF-κB and IRF3 signaling pathways, has been identified as a cellular partner of RSV N protein (174). The depletion of TAX1BP1 resulted in enhanced antiviral and inflammatory responses and restricted RSV replication. These results suggest that RSV inhibits the host’s innate immune response through TAX1BP1 recruited by the RSV N protein (174).

LLPS constitutes an indispensable part of virus antagonism against the host’s innate defense. Viral proteins interfere with host cell functions by disrupting cellular LLPS processes to minimize host antiviral responses. Additionally, the phase separation of viral components and formation of viral MLOs lead to spatial and compartmental re-localization of host cellular factors, thus precluding the normal antiviral responses of host factors and facilitating viral replication.

## 6 Therapeutic strategy targeting LLPS

As LLPS participates in multiple processes during virus infection, targeting the phase separation process may represent a new tactic for designing novel antiviral drugs. However, as both the host and virus share the LLPS system, which leads to double-edged sword effects, it remains to be seen whether LLPS needs to be upregulated or restricted when designing a treatment strategy. Thus, therapy development against infectious diseases necessitates targeting autophagy in a more selective way with specific molecular targets.

### 6.1 Antiviral therapeutic strategies toward LLPS multivalent interactions

Targeting protein-protein interaction has long been considered a challenging task, but the number of successful cases has increased rapidly in the past decade. Recent studies have discovered successful chemical probes that dissolve viral condensates through interaction with viral proteins engaging in phase separation. A phase modulator is an optical modulator that can be used to control the optical phase of a laser beam and may target RNA or protein IDRs directly. LLPS of RNA and protein is widely involved in the assembly of organelles, and viral assembly of SARS-CoV-2 is also known to depend on LLPS (175). Treatment with 1,6- hexanediol (an LLPS inhibitor) can inhibit the formation of N protein phase separation, which not only weakens TAK1 and IKKβ in the process of virus infection but can also inhibit the inflammatory response and inflammatory factor release caused by COVID-19 infection, thus representing a potential anti-inflammatory strategy of targeting phase separation for treating COVID-19 (173). However, 1,6-hexanediol is highly cytotoxic and arrests phosphatase and kinase activities. A nontoxic alternative, propylene glycol, could provide an alternative to separate rotavirus replication factors (176). Additionally, many viruses achieve immune escape by hijacking host antiviral factors, among which cyclophilin A (CypA) is of concern because it can be hijacked by HIV, hepatitis C virus, and SARS, among others. Through an in-depth study of its mechanism, CypA has been found to bind to protein kinase R (PKR), which affects its ability to detect viruses (177). Therefore, in *PKR* knockout cells by CRISPR/Cas9, cyclophilin inhibitors have been shown to have a weak ability to prevent virus replication. Antiviral drugs targeting CypA can be used to treat many incurable viruses (177, 178). Targeting SARS-CoV-2-N protein LLPS is considered to be a promising treatment strategy, which does not restrict viral assembly but heightens MAVS-dependent IFN induction (159). Wang and colleagues designed and synthesized interference peptides NIP I-V with a DRI conformation, which were found to inhibit the formation of the interaction region of SARS-CoV-2 N protein dimer by blocking different DDs. *In vitro* and *in vivo* analyses demonstrated that NIP-V could disrupt SARS-CoV-2-N protein LLPS to alleviate the N protein-mediated suppression of the innate antiviral responses. Targeting N-RNA condensation with gallic catechin gallate (GCG) could be a potential treatment for COVID-19. The GCG in green tea polyphenols can interact with the virus N protein, thus inhibiting LLPS, achieving the effect of inhibiting SARS-CoV-2 replication and reducing virus transmission (179). Nanoantibodies are also believed to neutralize SARS-CoV-2 by blocking receptor interactions and LLPS (180, 181).

### 6.2 Antiviral therapeutic strategies targeting LLPS *via* post-transcriptional modification

Post-translational modification (PTM) of LLPS phase-separated molecules is a critical factor affecting LLPS performance as PTMs can change the structure, charge, hydrophobicity, and other properties of phase-separated proteins. Several common PTMs (including phosphorylation, arginine methylation, arginine citrullination, acetylation, ubiquitination, and poly (ADP-ribosylation), could also regulate protein LLPS properties during viral infection, thus targeting PTMs of phase-separated proteins could contribute to anti-viral drug design (6, 20).

Small-molecule modulators of host kinases or phosphatases may regulate LLPS and serve as antiviral agents. Indeed, activators of the SR protein kinase 1 (SRPK1) may represent potential antivirals because the phosphorylation of the N protein SR region of SARS-CoV-2 could attenuate RNA-induced LLPS and viral RNA transcription (182). The RNA-binding protein TDP-43 is a key component of stress granules, and hyper-phosphorylated and ubiquitinated TDP-43 deposits act as endosomes in the brain and spinal cord of patients with motor neuron diseases (182). TDP-43 releases mtDNA into the cytoplasm through mPTP to activate signal transduction of the cGAS-STING pathway (183, 184). It has been demonstrated that cGAS and STING inhibitors can prevent inflammation induced by TDP-43. Acetylated TDP-43 colocalizes with SARS-CoV-2 N protein and simultaneously impairs the ability of TDP-43 to bind RNA (175). Ubiquitination promotes the aggregation of MAVS, and the SARS-CoV-2-N protein undergoes LLPS with RNA, which inhibits Lys63-linked poly-ubiquitination and suppresses the innate antiviral immune response (159). SIRT1-mediated DNA-binding domain (DBD) deacetylation of IRF3/IRF7 has been shown to inhibit LLPS and innate immunity, resulting in increased viral load and mortality in mice (118). Small-molecule inhibitors of protein acetyltransferase, such as C646, could be used to probe LLPS in viral infections (as detailed in **Table 2**) (185).

**Table 2:** Examples of antiviral therapy targeting phase separation.

	Target	Condensate mechanistic hypothesis	Function	Intended therapeutic effect targeting LLPS	
multivalent interactions	cGAS	DNA binding to cGAS induces the formation of liquid-like condensates	The formation of condensates can promote cGAS activity.	Streptavidin, a secreted protein from the bacterium Streptomyces avidinii, binds to cGAS to enhance cGAS–DNA interactions and promote LLPS of this complex.	(124, 156)
	STING	STING forms condensates with stacked endoplasmic reticulum membrane in the presence of an excess amount of cGAMP.	The STING condensates recruit the downstream signalling kinase TBK1.	STING agonist PC7A triggers STING condensate formation and stimulates the prolonged production of proinflammatory cytokines.	(127, 157)
post-transcriptional modification	TRIM25	RNA binding triggers LLPS of TRIM25	Recruits RIG-I to condensates and increases its ubiquitylation by TRIM25	Reintroduction of wild-type TRIM25.	(117)
	NEMO	Protein ubiquitination facilitate LLPS	Activate IKK complex.	Proteolysis-targeting chimeras and inhibitors of ubiquitin ligases is expected to be an additional effective strategy to treat neurodegenerative diseases.	(139, 158)
	MAVS	when SARS2-NP was in excess, the MAVS–MAVS was reported to form clusters in vivo and prion-like aggregates in vitro.	MAVS undergo LLPS and thus benefit antiviral signalling transduction.	Interfering peptide NIP-V targeting the DD disrupts SARS2-NP LLPS and thus enhances the innate antiviral response both in vitro and in vivo.	(159)
	IRF3/IRF7	SIRT1 deacetylates IRF3/IRF7 and induces LLPS of IRF3 with interferon (IFN)-stimulated response element DNA.	IRF3 LLPS compartmentalized IRF7 in the nucleus, thereby stimulating type I IFN (IFN-I) expression.	A natural phytoalexin compound, resveratrol (SRT501) and a chemically synthetic compound SRT2183 reinforces SIRT1 activity.	(160, 161)

### 6.3 Heat-treatment-based therapeutic strategies against viral LLPS condensation

In addition to directly targeting the formation of LLPS, it is also possible to alter LLPS environmental factors to regulate the state of the target protein LLPS. The RNA binding domains (RBDs) of SARS-CoV-2 N-protein, RBD1 and RBD2, interact with different dsRNA. The addition of dsRNA reduces the condensation temperature, dependent on the RBD2 interaction, and regulates the inhibition of translation (186). It has been reported that in HPV-infected cells, the early protein 7 (E7) mRNA of the virus is modified by m6A and stabilized by the cell m6A reader IGF2BP1. Heat treatment promotes the aggregation of IGF2BP1 in the presence of m6A-modified E7 mRNA to form different heat-induced m6A E7 mRNA-IGF2BP1 particles, which are decomposed by the ubiquitin-proteasome system, and reverse HPV-related carcinogenesis *in vitro* and *in vivo* (187). During continued heat stress, the LLPS characteristics of heat-induced IGF2BP1 condensate suggested a liquid-to-solid phase transition of IGF2BP1 puncta. Several studies have suggested that high temperature and high humidity environments are conducive to reducing the transmission rate of COVID-19 (187). SGs are very sensitive to oxidative stress, osmotic stress, heat shock stress, and other cell stress conditions. FUS protein, a component of stress granules in cells, can form highly reversible amyloid fibrin through LLPS. FUS undergoes LLPS condensation with the SARS-CoV-2 N protein and may be involved in the host-mRNA processing of N; therefore, temperature may be related to some translation mechanisms around SG during stimulation (188). These studies show that controlling environmental factors, such as temperature, is an effective strategy to inhibit viral LLPS and virus replication, thus exploiting LLPS through adjusting the environmental factors might present tremendous potential as an adjuvant systematic therapy.

## 7 Discussion

The application of LLPS during viral replication and host antiviral immunity has been greatly extended in the last several years. Several previous reports have observed viral structures such as IBs, Negri bodies from negative strand RNA viruses, and viral replication compartments from DNA viruses, which were recently regarded to arise from LLPS processes. These membraneless cellular viral factories formed by virus components contribute to viral replication, capsid assembly, virion generation, and immune restriction. In contrast, the host also uses the LLPS system to ensure the effective elimination of invading microorganisms. However, numerous questions remain unanswered regarding viral and host components of LLPS condensates. Further studies are required to investigate the detailed mechanisms and driving forces of the formation of host and viral LLPS condensation, the correlation between host and viral LLPS, the overall properties of host and viral molecular condensates, and the complete composition in viral LLPS MLOs. Considering the lack of a comprehensive understanding of host and viral LLPS condensates during infection, antiviral interventions targeting host and viral LLPS condensates are still at a very early stage. How to effectively restrict viral LLPS to limit viral replication and modulate LLPS of host antiviral components to ensure appropriate immune responses, even the precise control of specific molecules, remains a long-standing challenge in the implementation of phase separation-based therapies.

## Author contributions

YW and JC conceived and designed the manuscript. SY, WS and YW drafted the manuscript. JH drew the plots. The manuscript was written by SY, WS and YW. All authors contributed to the article and approved the submitted version.
